# Another Advance Is Necessary

**Published:** 1894-03

**Authors:** 


### Editorial.

### ANOTHER ADVANCE IS NECESSARY.

It was with many misgivings as to the results when, in 1884, at the request of the dean of the Baltimore College of Dental Surgery, four men met together in Philadelphia, representatives of as many colleges, to consult in regard to the condition of dental education in this country, and whether it were feasible by concerted action to make any improvements in the unsatisfactory state which then existed.

The status of dental education at that period was about as bad as it could possibly be. The large majority of the schools were acting under a nominal two years, with courses of from four to five months. The so-called rule of "five years' practice," admitting students to the senior year who could present evidence of having had five years' practical experience, was in full force in the large majority of colleges. The results that had followed the adoption of this rule had become a professional scandal, as it was a notorious fact that a very large proportion thus admitted never had had the practice required.

Each of the schools was a law unto itself, and naturally resisted any invasion of the privileges accorded by the State charters under which they all operated, and any effort looking towards a change for the better seemed hopeless.

It is not surprising, then, that the self-appointed consultants looked gloomily at the present condition of professional training, and could not anticipate any great gain from the effort proposed. Would any considerable number of the colleges join in such a movement was the question uppermost in their minds. It was worth a trial, and the result of that conference was the issuing of the now historical call for a "meeting of Faculties (or their representatives) of all the dental colleges in the United States at the Sturtevant House, New York City, Monday, August 4, 1884, for the purpose of adopting a uniform standard of graduation, etc."

This meeting took place, and it was not only gratifying, but promised well for the proposed conference, that eleven colleges and departments of universities were represented.

No precedents existed for such an organization, and when the representatives met, it was felt that if it were possible to effect a permanent association the laws governing it must be based on experience. Legislation in advance of this was not deemed practicable, and hence resulted one of the simplest constitutions, perhaps, ever adopted by an organized body.

The wisdom of this arrangement has been made clearly apparent by the lapse of time, but in this conference they builded "better than they knew."

It was very evident from the earnestness manifested at this meeting that the representatives present, who, with one exception, took part in the permanent association, were prepared for positive measures looking towards reform. It was, therefore, not surprising that the first resolution adopted became the basis of a series of working rules for the government of this body. This was apparently a very simple one, but meant very much at that period. It was that "after the close of the session 1884–85 students in dental colleges shall be required to attend two full regular courses of lectures in separate years." The adoption of this under then existing circumstances was a bold move, but its very boldness insured its immediate acceptance by nearly all the colleges then outside of the association. It was clearly evident that this organization meant to work up to a higher standard, and that all schools failing to follow must necessarily have the stigma of imperfection fastened upon them.

From this period the National Association of Dental Faculties became a power in the dental educational world, and when, subsequently, the National Association of Boards of Examiners was established, the results became more pronounced and the anxiety of outside colleges to fall in line with this work more decided.

Nearly ten years have passed since the time of that first meeting. Not one of the original number could have foreseen the remarkable results that followed that effort.

The scandalous rule of "five years' practice" was at once abolished. A strict surveillance of the actions of the various schools composing the membership was adopted, to be executed by an appropriate committee, and, while it may be possible that the rules are occasionally violated, it is gratifying to feel that this must be very exceptional.

The next serious move was the establishment of three years of study and attendance at the schools, together with a course of not less than five months to be taken in separate years. This, while not satisfactory to those departments having from seven to nine months, it was the best attainable at the time.

When it is remembered that the larger number of medical schools at that period had courses not over two years, it is not surprising that the weaker dental colleges at first objected to this extension, and it required several years of argument and persuasion before a decision could be reached. In the mean time many of the medical colleges had advanced the time. This, together with the fact that pressure was brought to bear by the profession at large through conventions and boards of examiners, led to its final adoption in 1889, to take effect in 1891–92.

The result of this move, after now nearly three years of trial, has been more satisfactory than the most sanguine could have predicted. While there has been a slight falling off in students in the first and second years, the aggregate has increased, and from a financial point of view the schools are better off to-day than at any former period. This is so marked that it ought to demonstrate to the most pessimistic that any further reasonable advance in the standard will not only prove of lasting benefit to colleges and students, but will increase the stability of the institutions.

There are three important advances yet to be made, and we approach the consideration of these with some hesitation. Not but that they are all necessary and in the line of progress, but it may be a question whether, in the case of two, the time has yet come for their adoption. Progress made by forced marches is not always the best, changes should be approached carefully and with due consideration of all the interests involved.

We will, therefore, first consider the one that seems to us feasible and worthy of immediate adoption.

As far back as 1889 an effort was made to have a resolution passed lengthening the course of each college to at least seven months. This was at that time laid upon the table, as it was not regarded advisable to add too much to the then anticipated three years' course.

This change to three years has demonstrated fully its own value, but there still remains a lack of uniformity in colleges and renders it impossible for the eight and nine months departments to accept those of five. Aside from this, perhaps a minor point, the one term of five months is too much in the line of past mistakes. While it is nominally five months, it really means not more than three and a half, for it must be evident that the first two weeks in October must be given to organizing in all well-conducted colleges, and that the last month is altogether broken up by examinations. This makes but ten and a half months of actual work for the three years. It may be argued that the spring term lengthens this, but it is notorious that this is not obligatory, and but few avail themselves of its manifold advantages.

The time has certainly come when a change can be made in this, and it can be accomplished without injury to any school the present year. It is certainly to be hoped that the National Association of Dental Faculties will at the meeting in August, *extend all the courses to at least seven months.*

The other two changes are first in the preliminary examinations. It is questionable whether the profession are ready to advance this beyond what is regarded as a good English education, and we are not sure that it would be advisable at the present time, but it is an additional reform that must come. We can have no sympathy with the methods adopted in England and on the Continent in this respect, and do not believe that the high standard there required can ever be adopted in this country as far as dentistry is concerned. In order to meet its demands a young man's best years are sacrificed to the attainment of information which, while in itself of great value, is utterly useless in a practical profession such as ours must ever remain. The change, if any be made, must be made to a slightly higher standard.

The colleges should look forward to the final and, perhaps, the last great advance, the extension to four courses of eight months each in separate years. This accomplished, it is believed dentistry will be founded on a rock, and must from that time rear a superstructure in which all the parts will advance steadily to something approaching perfection. If this be attained, the work of the National Association of Dental Faculties will have been mainly accomplished.

